# Meta-analysis of set-based multiple phenotype association test based on GWAS summary statistics from different cohorts

**DOI:** 10.3389/fgene.2024.1359591

**Published:** 2024-09-05

**Authors:** Lirong Zhu, Shuanglin Zhang, Qiuying Sha

**Affiliations:** Department of Mathematical Sciences, Michigan Technological University, Houghton, MI, United States

**Keywords:** meta-analysis, joint analyses of multiple phenotypes, set-based association tests, GWAS summary statistics, phenotype simulator, multiple GWAS cohorts

## Abstract

Genome-wide association studies (GWAS) have emerged as popular tools for identifying genetic variants that are associated with complex diseases. Standard analysis of a GWAS involves assessing the association between each variant and a disease. However, this approach suffers from limited reproducibility and difficulties in detecting multi-variant and pleiotropic effects. Although joint analysis of multiple phenotypes for GWAS can identify and interpret pleiotropic loci which are essential to understand pleiotropy in diseases and complex traits, most of the multiple phenotype association tests are designed for a single variant, resulting in much lower power, especially when their effect sizes are small and only their cumulative effect is associated with multiple phenotypes. To overcome these limitations, set-based multiple phenotype association tests have been developed to enhance statistical power and facilitate the identification and interpretation of pleiotropic regions. In this research, we propose a new method, named Meta-TOW-S, which conducts joint association tests between multiple phenotypes and a set of variants (such as variants in a gene) utilizing GWAS summary statistics from different cohorts. Our approach applies the set-based method that Tests for the effect of an Optimal Weighted combination of variants in a gene (TOW) and accounts for sample size differences across GWAS cohorts by employing the Cauchy combination method. Meta-TOW-S combines the advantages of set-based tests and multi-phenotype association tests, exhibiting computational efficiency and enabling analysis across multiple phenotypes while accommodating overlapping samples from different GWAS cohorts. To assess the performance of Meta-TOW-S, we develop a phenotype simulator package that encompasses a comprehensive simulation scheme capable of modeling multiple phenotypes and multiple variants, including noise structures and diverse correlation patterns among phenotypes. Simulation studies validate that Meta-TOW-S maintains a desirable Type I error rate. Further simulation under different scenarios shows that Meta-TOW-S can improve power compared with other existing meta-analysis methods. When applied to four psychiatric disorders summary data, Meta-TOW-S detects a greater number of significant genes.

## 1 Introduction

Genome-wide association study (GWAS) is typically employed to identify an individual genetic variant associated with a specific phenotype. However, in cases where causal variants have weak effects on the trait and it is challenging to detect these variants, set-based tests are employed to identify the joint effects of multiple variants (multi-variants) on a particular phenotype (Lin et al., 2022; Huang et al., 2011; Liu et al., 2010; Neale and Sham, 2004). Compared to single-variant approaches, set-based tests can help to reduce the number of genome-wide association tests and are effective when a causal variant is unobserved or multiple causal variants are present (Lin et al., 2022). Consequently, set-based tests have been applied in gene-set analyses of common variants and rare variants (Liu et al., 2014). For instance, both weighted and unweighted burden tests have a good performance when variants in a gene affect a phenotype in the same direction (Morris and Zeggini, 2010). The sequence kernel association test (SKAT) is a score-based variance-component test that accommodates variants in a gene with opposite effects on a phenotype (Wu et al., 2011). However, the performance of the approaches mentioned above depends on the weighting scheme, such as the MAF-based weighting scheme that up-weights the contribution of rare variants and down-weights that of common variants. This weighting scheme may lead to a loss in power when common variants in the region are associated with the phenotype (Dutta et al., 2019b; Dutta et al., 2019a). The optimal strategy for grouping or weighting genetic variants depends on the unknown genetic architecture of each phenotype and each variant. TOW for Testing the effect of an Optimal Weighted combination of variants in a set is a powerful method to increase power by assigning optimal weights to genetic variants (Sha et al., 2012).

Many complex phenotypes are influenced by multiple genetic variants, each with a small effect size. Set-based tests that consider a single phenotype might not capture the collective effects of these variants, leading to reduced power. Alternatively, cross-phenotype tests that aggregate associations in multiple phenotypes can substantially improve power over single phenotype-based methods (Dutta et al., 2019b; Dutta et al., 2019a). Meta-analysis of multiple phenotypes, using GWAS summary statistics, is a practical approach to increase power by increasing sample sizes and aggregating variants with small effect sizes to detect more significant pleiotropic genes (Cirulli et al., 2020; Panagiotou et al., 2013). Detecting the pleiotropic genes can provide insights into biological mechanisms influencing complex human phenotypes. The challenge in meta-analysis is that there is no uniformly most powerful (UMP) test. The power depends on signal directions and between-phenotype correlation. To boost analysis power, several methods have been proposed for GWAS multiple phenotype analysis. For instance, Fisher’s method of combining independent *p*-values has been extended to dependent univariate tests (Li et al., 2014). However, the *p*-value approximations of these tests are not accurate for small significance levels often required by GWASs. The minimum of the *p*-values (MinP) of multiple phenotypes has been proposed as a testing statistic (Conneely and Boehnke, 2007). And this test is powerful when a gene affects only a very small number of multiple phenotypes, but is less powerful in the presence of denser signals (Liu and Lin, 2018). The aggregated Cauchy association test (ACAT) is a flexible and computationally efficient *p*-value combination method that boosts power under various genetic architectures (Liu and Xie, 2019; Liu et al., 2019). Thus, in this article, we applied this approach to combine *p*-values of set-based tests from multiple cohort studies. Most importantly, the Cauchy combination is an extremely fast omnibus testing procedure that performs multiple testing adjustments analytically and applies to the combination of any tests (Li et al., 2023; Li et al., 2022; Li et al., 2020).

Meta-analysis is a promising approach to detecting a series of gene-phenotype associations that would have remained undetected by a participating cohort alone. In addition, a meta-analysis based on GWAS summary statistics simplifies data sharing, keeping sensitive individual data at the cohort level and sharing only non-sensitive summary data (Lin et al., 2022). For instance, Meta-MultiSKAT performs a variance component test and uses summary statistics to test for association between multiple continuous phenotypes and variants in a region. However, the *p*-value calculation of Meta-MultiSKAT relies on the normality assumption of the score vector and this assumption may be violated in the meta-analysis (Dutta et al., 2019b; Dutta et al., 2019a). MetaUSAT is a novel unified association test of multiple traits with only a single genetic variant, and the test statistic is dominated by a predefined parameter weight (Ray and Boehnke, 2018). In the cross meta-analysis, the overlapping subjects can induce a correlation between the summary statistics and inflate the false discovery rate of meta-analyses. Method FOLD is proposed to account for overlapping subjects at the summary statistics level using a split prior which categorizes subjects based on their contributions to the final statistic (Kim et al., 2017). However, it is designed for qualitative phenotypes and is difficult to obtain splitting prior if the numbers of cases and controls in any of the GWAS cohorts are missing. Here we propose a new approach, Meta-TOW-S, which conducts joint association tests between multiple phenotypes and genetic variants within a gene, utilizing GWAS summary statistics from diverse GWAS. Our approach applies set-based tests using an optimal weighted combination of variants and accounts for sample size differences across different GWAS by employing the Cauchy combination method. Meta-TOW-S combines the advantages of set-based tests and multi-phenotype modeling, exhibiting computational efficiency and enabling analysis across multiple phenotypes while accommodating overlapping samples from different cohorts.

To evaluate the performance of Meta-TOW-S, we need to mimic GWAS summary data from different cohorts. Thus, we also develop a phenotype simulator package that encompasses a comprehensive simulation scheme capable of modeling multiple phenotypes with multiple underlying genetic loci, intricate noise structures, and diverse correlation patterns among the phenotypes. The R package for the phenotype simulator is available on GitHub (https://github.com/Julia-lirong/PheGen). Furthermore, we evaluate the performance of our method using simulation studies and compare the power of our method with the power of three existing methods which integrate Burden (Morris and Zeggini, 2010), SKAT (Wu et al., 2011), and VEGAS (Liu et al., 2010) with Cauchy combination (Liu and Xie, 2019) to detect pleiotropic effects. Our simulation studies validate that Meta-TOW-S maintains a desirable Type I error rate and enhances power across various simulation scenarios compared with other existing meta-analysis methods. We also apply Meta-TOW-S to four psychiatric disorders summary data which are available from the Psychiatric Genomics Consortium (PGC) (Sullivan et al., 2018). The real data analyses demonstrate that Meta-TOW-S outperforms other comparison methods by detecting a greater number of significant genes.

## 2 Materials and methods

Consider K phenotypes from K GWAS cohorts with sample sizes $n_{1},n_{2},\ldots ,n_{K}$ that are subject to $N=\underset{k=1}{\sum^{K}}n_{k}$ . For the $k^{th}$ cohort, suppose that the GWAS summary statistics $S_{k}={\left(Z_{k}^{1},Z_{k}^{2},\ldots ,Z_{k}^{M}\right)}^{T}$ are the Z-scores of M genetic variants in a genomic region. We assume all cohorts share the same genetic variants in the specific region. $N_{s}$ is the number of overlapping subjects among all cohorts, where $N_{s}\leq \min\left\{n_{1},n_{2},\ldots ,n_{K}\right\}$ .

### 2.1 Meta-TOW-S

For the $k^{th}$ cohort, we suppose that $G_{k}$ is a $n_{k}\times M$ matrix of genotypes in the interested genomic region (gene or pathway), and $y_{k}$ is a $n_{k}\times 1$ vector of phenotypes (either a quantitative or qualitative phenotype). In TOW (Sha et al., 2012), the generalized linear regression model with the fixed effect is used to link the phenotype and genotypes. The statistic model is defined as $g\left(E\left(y_{k}|G_{k}\right)\right)=\beta_{k0}+G_{k}w_{k}\beta_{k}$ , where $w_{k}$ is the vector of weights for the M genetic variants; $w_{k}={\left(w_{k1},\ldots ,w_{kj},\ldots ,w_{kM}\right)}^{T}$ , and $w_{kj}$ is the weight assigned to the $j^{th}$ variant in the $k^{th}$ cohort. $\beta_{k}$ is the effect size of the weighted combination of genetic variants $G_{k}w_{k}$ on the phenotype $y_{k}$ . Under the null hypothesis of no association between the variants in the region and the $k^{th}$ phenotype, we test $H_{0}∶\beta_{k}=0$ . The score test statistic is given by $T_{k}=n_{k}\frac{w_{k}^{T}G_{k}^{T}P_{0}{y_{k}y}_{k}^{T}P_{0}G_{k}w_{k}}{y_{k}^{T}P_{0}{y_{k}w_{k}^{T}G}_{k}^{T}P_{0}G_{k}w_{k}}=\frac{u_{k}^{T}w_{k}w_{k}^{T}u_{k}}{w_{k}^{T}\Sigma_{k}w_{k}}$ , where $P_{0}=I_{n_{k}}-\frac{1}{n_{k}}1_{n_{k}}1_{n_{k}}^{T}$ , $u_{k}=G_{k}^{T}P_{0}y_{k}/\sqrt{y_{k}^{T}P_{0}y_{k}/n_{k}}$ , $\Sigma_{k}=G_{k}^{T}P_{0}G_{k}$ , $1_{n_{k}}$ represents a $n_{k}\times 1$ vector containing all ones, and $I_{n_{k}}$ is a $n_{k}\times n_{k}$ identity matrix. Under the null hypothesis, $u_{k}$ follows a multivariate normal distribution with mean vector 0 and covariance matrix $\Sigma_{k}$ . The TOW method obtains the optimal weights ${w_{k}}^{*}$ by maximizing the score test statistic $T_{k}$ using the Cauchy-Schwartz inequality. Specifically, we have the form $T_{k}=\frac{u_{k}^{T}w_{k}w_{k}^{T}u_{k}}{w_{k}^{T}\Sigma_{k}w_{k}}=\frac{u_{k}^{T}{E^{-1/2}E^{1/2}w}_{k}w_{k}^{T}E^{-1/2}E^{1/2}u_{k}}{w_{k}^{T}\Sigma_{k}w_{k}}\leq \frac{\langle E^{-\frac{1}{2}}u_{k},E^{-\frac{1}{2}}u_{k}\rangle \langle E^{\frac{1}{2}}w_{k},E^{\frac{1}{2}}w_{k}\rangle }{w_{k}^{T}\Sigma_{k}w_{k}}=\frac{w_{k}^{T}Ew_{k}}{w_{k}^{T}\Sigma_{k}w_{k}}u_{k}E^{-1}u_{k}=cu_{k}E^{-1}u_{k}$ , with the equality when $w_{k}\propto E^{-1}u_{k}$ , where E is any M×M positive definite matrix, and $c=\frac{w_{k}^{T}Ew_{k}}{w_{k}^{T}\Sigma_{k}w_{k}}$ is a constant. Based on Yan’s work (Yan, 2022), $T_{k}=cu_{k}E^{-1}u_{k}$ is a quadric term with the asymptotical distribution of weighted sum of Chi-squares. We assume $\Sigma_{k}$ is full rank and let $E=\Sigma_{k}$ . Then the optimal weight is obtained with the form ${{w_{k}}^{*}=\Sigma_{k}}^{-1}u_{k}$ . Using the optimal weights, the test statistic of TOW is given by $T_{k}^{\left(TOW\right)}=u_{k}^{T}\sum_{k}^{-1}u_{k}=n_{k}\frac{y_{k}^{T}P_{0}G_{k}\Sigma_{k}^{-1}G_{k}^{T}P_{0}y_{k}}{y_{k}^{T}P_{0}y_{k}}$ . Under the null hypothesis, $T_{k}^{\left(TOW\right)}$ asymptotically follows a Chi-square distribution with a degree of freedom M , that is $T_{k}^{\left(TOW\right)}∼\chi_{M}^{2}$ (Yan, 2022; Sha et al., 2012).

Inspired by the idea proposed in this project (Svishcheva et al., 2019), Yan (2022) rewrite the test statistic $T_{k}^{\left(TOW\right)}$ using GWAS summary statistics. For the $k^{th}$ cohort, the Z score of the M genetic variants in a region can be written as $S_{k}={\left(Z_{k}^{1},\ldots ,Z_{k}^{M}\right)}^{T}=\frac{V_{k}^{-1}G_{k}^{T}P_{0}y_{k}}{\sqrt{y_{k}^{T}P_{0}y_{k}/n_{k}}}$ , where $V_{k}$ is an M×M diagonal matrix of the square roots of genotypic variances of the M variants. We assume that under the null hypothesis, $S_{k}$ follows the multivariate normal distribution $N\left(0,U\right)$ , where U is an M×M matrix of correlations between the genotypes of these variant and $U=\frac{V_{k}^{-1}G_{k}^{T}{P_{0}G}_{k}V^{-1}}{n_{k}}$ . Then the test statistic of TOW based on individual data can be written as $T_{k}^{\left(TOW\right)}=n_{k}\frac{y_{k}^{T}P_{0}G_{k}\Sigma_{k}^{-1}G_{k}^{T}P_{0}y_{k}}{y_{k}^{T}P_{0}y_{k}}=S_{k}^{T}U^{-1}S_{k}$ . This test statistic is called $T_{TOW-S}\left(S_{k}\right)$ and $T_{TOW-S}\left(S_{k}\right)∼\chi_{M^{\prime }}^{2}$ asymptotically, where $M^{\prime }$ is the number of variants left after correlation pruning (Svishcheva et al., 2019). We can estimate U using a reference sample of genotypes from the same population, such as 1,000 Genome phase 3 if individual genotype data are not available.

Consider K phenotypes from different GWAS cohorts, we denote $p_{k}$ as the *p*-value of $T_{TOW-S}$ for the $k^{th}$ GWAS cohort, where k=1,…,K . Then to detect the association between genetic variants in this region and multiple phenotypes, we define the Cauchy combination test statistic as $T_{C}=\underset{k=1}{\sum^{K}}\nu_{k}\tan\left\{\left(0.5-p_{k}\right)\pi \right\}$ , where the weight is defined as $\nu_{k}=\frac{n_{k}}{N}$ and $T_{C}$ has a standard Cauchy distribution under the null (Liu and Xie, 2019). Here we assigned more weight to a large GWAS cohort because a GWAS cohort with a large sample size carries more information than a smaller GWAS cohort (Zhu et al., 2015).

### 2.2 Comparison with other set-based association tests

Versatile set-based association study (VEGAS): For a specific region with M genetic variants in the $k^{th}$ GWAS summary study, the test statistic of VEGAS is the sum of all squared Z-scores which is $T_{Vegas}\left(S_{k}\right)=S_{k}^{T}S_{k}$ (Liu et al., 2010). Under the null hypothesis, $T_{Vegas}\left(S_{k}\right)$ asymptotically follows a mixture of chi-square distribution. To obtain the *p*-value of VEGAS, several methods have been proposed, such as numerical inversion of the characteristic function (Liu et al., 2009), Davies method (Davies, 1980), or Saddlepoint approximation (Kuonen, 1999).

SKAT and Burden: For the $k^{th}$ cohort, we consider the generalized linear model with the random effect, we have $g\left(E\left(y_{k}|G_{k}\right)\right)=\beta_{k0}+G_{k}\beta_{k}$ , where $\beta_{k}$ is a vector of effect size of the M variants which is assumed to follow a normal distribution $N\left(0,\tau_{k}^{2}I\right)$ under the null hypothesis, where $\tau_{k}^{2}$ is the variance component of the phenotype explained by the M variants. To test the association between the M genetic variants in a region and the phenotype, Burden and SKAT test the null hypothesis $H_{0}∶\tau_{k}^{2}=0$ against $H_{a}∶\tau_{k}^{2}>0$ (Svishcheva et al., 2019). For the $k^{th}$ GWAS cohort, the burden test statistic using GWAS summary statistics can be written as $Q_{BT}\left(S_{k}\right)={\left(S_{k}^{T}W1_{M}\right)}^{2}$ , where W is a M×M diagonal matrix with $W_{jj}=1/\sqrt{MAF_{j}\left(1-MAF_{j}\right)}$ , and $MAF_{j}$ is the minor allele frequency for the $j^{th}$ variant (Lee et al., 2013). Under the null hypothesis, $Q_{BT}$ follows a scaled Chi-square distribution with one degree of freedom. In SKAT, the test statistic based on GWAS summary statistic is defined as $Q_{SKAT}\left(S_{k}\right)={S_{k}^{T}\text{WW}}^{T}S_{k}$ , where W is a M×M diagonal matrix of weights with $W_{jj}=Beta\left(MAF_{j};a_{1},a_{2}\right)$ . The beta distribution density function has pre-specified parameters $a_{1}$ , $a_{2}$ and $MAF_{j}$ . To obtain the *p*-value of Burden and SKAT, several methods have been proposed, such as numerical inversion of the characteristic function (Liu et al., 2009), Davies method (Davies, 1980), or Saddlepoint approximation (Kuonen, 1999).

Let $p_{k}$ be the *p*-value of the $k^{th}$ cohort study for k=1,…,K based on the three comparison methods Vegas, Burden, and SKAT. We use the same strategy to detect the association between multiple phenotypes in different GWAS cohorts and genetic variants in a region by employing the Cauchy combination (Liu and Xie, 2019). And we designate these three methods as Meta-Vegas, Meta-Burden, and Meta-SKAT, respectively.

## 3 Simulation studies

### 3.1 Phenotype simulator

Suppose we have K cohorts with sample sizes $n_{1},n_{2},\ldots ,n_{K}$ , respectively. For the $k^{th}$ cohort, the phenotype is modeled by a linear model $y_{k}=G_{k}\beta_{k}+\varepsilon_{k}$ , where $G_{k}$ are matrices of genotypes with columns standardized to mean zero and variance 1, with dimension $n_{k}\times M$ . $y_{k}$ is a $n_{k}\times 1$ vector of standardized phenotypes with mean zero and variance 1. $\beta_{k}$ is the vector of genotypes effect sizes in the specific gene and $\varepsilon_{k}$ is the vector of residuals representing environmental effects and non-additive genetic effects for the $k^{th}$ cohort. For each cohort, we assume that all genotype effect sizes are drawn with equal variance for all causal variants in a gene (Lee et al., 2014). Then for all K cohorts, we suppose that the matrix of effect size $\left(\beta_{1},\beta_{2},\ldots ,\beta_{K}\right)$ has mean zero and covariance matrix $Var\left[\left(\beta_{1},\beta_{2},\ldots ,\beta_{K}\right)\right]=\frac{1}{M}\left[\begin{array}{cc} \begin{array}{ccc} {\;h}_{1}^{2}I & \rho_{g_{12}}I & \ldots \end{array} & \rho_{g_{1K}}I\\ \begin{array}{ccc} \begin{array}{c} \rho_{g_{12}}I\\ \ldots \\ \rho_{g_{1K}}I \end{array} & \begin{array}{c} h_{2}^{2}I\\ \ldots \\ \rho_{g_{2K}}I \end{array} & \begin{array}{c} \ldots \\ \ldots \\ \ldots \end{array} \end{array} & \begin{array}{c} \rho_{g_{2K}}I\\ \ldots \\ h_{K}^{2}I \end{array} \end{array}\right]$ , where $\rho_{g_{ij}}$ is the genetic covariance on two phenotypes i and j on shared individuals, $h_{k}^{2}$ is the heritability explained by the variants in a region for the $k^{th}$ phenotype. The residuals ${\left(\varepsilon_{1},\varepsilon_{2},\ldots ,\varepsilon_{K}\right)}^{T}$ has mean zero and covariance matrix $Var\left[{\left(\varepsilon_{1},\varepsilon_{2},\ldots ,\varepsilon_{K}\right)}^{T}\right]=\left[\begin{array}{cc} \begin{array}{ccc} \left({1-h}_{1}^{2}\right)I & \rho_{e_{12}}I & \ldots \end{array} & \rho_{e_{1K}}I\\ \begin{array}{ccc} \begin{array}{c} \rho_{e_{12}}I\\ \ldots \\ \rho_{e_{1K}}I \end{array} & \begin{array}{c} \left({1-h}_{2}^{2}\right)I\\ \ldots \\ \rho_{e_{2K}}I \end{array} & \begin{array}{c} \ldots \\ \ldots \\ \ldots \end{array} \end{array} & \begin{array}{c} \rho_{e_{2K}}I\\ \ldots \\ \left(1-h_{K}^{2}\right)I \end{array} \end{array}\right]$ , where $\rho_{e_{ij}}$ is the covariance of non-genetic effects on the $i^{th}$ and $j^{th}$ phenotypes among shared individuals. Then, the phenotypic correlation for cohort i and cohort j among the $N_{s}$ overlapping samples is $\rho_{ij}=\rho_{g_{ij}}+\rho_{e_{ij}}$ (Lemma). For these $N_{s}$ overlapping individuals, all cohorts share the same genotype data.

Next, to generate genotypes for individuals in a cohort, we employ the calibration coalescent model (COSI) to generate 10,000 haplotypes for a region of approximately 200 kbps, mimicking the LD structure found in individuals of European ancestry (He et al., 2017). We randomly select regions of 10 kbps in length, which encompass approximately 100 genetic variants, and utilize the simulated haplotypes to create genotypes for the variant sets. Among these genetic variants, we specifically designate 10% as causal variants, comprising 60% rare variants and 40% common variants, respectively. Subsequently, we generate the genetic component $\left(\beta_{1},\beta_{2},\ldots ,\beta_{K}\right)$ and non-genetic component ${\left(\varepsilon_{1},\varepsilon_{2},\ldots ,\varepsilon_{K}\right)}^{T}$ based on the distribution described above. Specifically, we fix the phenotypic correlation between each pair of phenotypes. The phenotype correlation among the overlapping individuals is influenced by both genetic and non-genetic covariance, as proven in Lemma in the Supplementary Material. We allocate 80% of the phenotypic correlation to genetic covariance and 20% to non-genetic covariance. Finally, we generate K quantitative phenotypes in different cohorts using the additive model $y_{k}=G_{k}\beta_{k}+\varepsilon_{k}$ , $k\in \left\{1,2,\ldots ,K\right\}$ .

### 3.2 Simulation

To set up a multi-cohort scenario, we generated individuals for multiple cohorts with different sample sizes but the same overlapping sample size for simplicity. To achieve a normally distributed input for the association test between gene and phenotype within each cohort, a rank-based inverse-normal transformation to the residuals of each phenotype was performed. In simulations, we access the performance of Meta-TOW-S with compared methods Meta-Vegas, Meta-Burden, and Meta-SKAT. We design different patterns of phenotypes by varying the correlation of phenotypes, sample sizes, and overlapping samples.

## 4 Results

### 4.1 Type I error rates

To evaluate the Type I error rates of Meta-TOW-S, we first simulate 1,000 regions, each encompassing approximately 100 genetic variants, reflecting the LD structure observed in individuals of European ancestry. We then replicate $10^{5}$ times to generate the phenotypes under the null hypothesis of no genetic contribution to any of the three traits, that is β=0 . Then we simulate $10^{8}$ datasets to estimate the Type I error rates at nominal significance levels $\alpha =2.5*10^{-6},10^{-5},10^{-4}$ . We generate three phenotypes with different sample sizes. In the first situation, the sample sizes for the three phenotypes are equal, with a ratio of 1000:1000:1000 . In the second scenario, the sample sizes are unequal, with a ratio of 2000:1000:500 . For simplicity, the number of overlapping individuals between any two phenotypes is kept constant at 500 . The phenotypic correlation between any two phenotypes among the overlapping individuals is set to be either ρ=0.5 or ρ=1 . A correlation of 1 indicates simulation of mimicking one phenotype but form different cohorts

For Type I error evaluations, we use the significance levels $\alpha =2.5*10^{-6},10^{-5},10^{-4}$ . Table 1 summarizes the estimated Type I error rates of the four tests based on different settings. We can see from Table 1, that the Type I error rates of all tests are all within the estimated 95% confidence intervals in most situations indicating that the Type I error rates of the four tests are well controlled at the nominal significance levels.

**TABLE 1:** Type I error estimates of the four tests. Each entry represents the Type I error rate estimated by the proportions of *p*-values less than α with $10^{8}$ simulations. The phenotypic correlation between any pair of phenotypes is set to be either ρ=0.5 or ρ=1 . The sample sizes of the three cohorts are $N_{1}∶N_{2}∶N_{3}=1000∶1000∶1000$ and $N_{1}∶N_{2}∶N_{3}=2000∶1000∶500$ .

Correlation between phenotypes	Sample size in each cohort	Number of overlapped samples	Significance level	Meta-TOW-S	Meta-Vegas	Meta-SKAT	Meta-burden
0.5	1,000:1,000:1,000	500	1.00E-04	1.03E-04	9.21E-05	8.81E-05	1.02E-04
			1.00E-05	9.66E-06	9.53E-06	7.88E-06	1.03E-05
			2.5E-06	2.27E-06	2.74E-06	2.21E-06	2.33E-06
0.5	2,000:1,000:500	500	1.00E-04	9.94E-05	9.15E-05	9.04E-05	9.80E-05
			1.00E-05	9.45E-06	8.63E-06	8.47E-06	9.92E-06
			2.5E-06	3.05E-06	2.42E-06	2.32E-06	2.67E-06
1	1,000:1,000:1,000	500	1.00E-04	1.11E-04	9.09E-05	8.72E-05	1.00E-04
			1.00E-05	9.82E-06	8.31E-06	7.69E-06	1.02E-05
			2.5E-06	2.40E-06	2.23E-06	1.74E-06	2.64E-06
1	2,000:1,000:500	500	1.00E-04	8.97E-05	9.10E-05	8.79E-05	1.02E-04
			1.00E-05	8.17E-06	8.36E-06	7.92E-06	1.02E-05
			2.5E-06	1.93E-06	2.45E-06	1.93E-06	2.61E-06

### 4.2 Power comparisons

**FIGURE 1:**
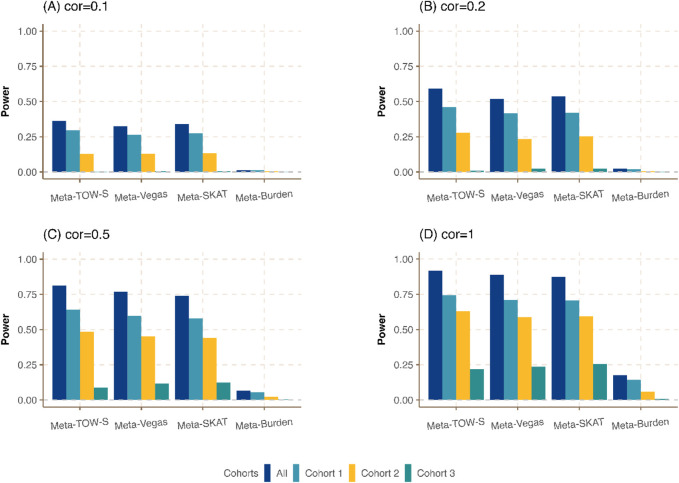
Power comparison of Meta-TOW-S, Meta-Vegas, Meta-SKAT, and Meta-Burden with single phenotype-based set-level tests at the significance level $\alpha =2.5\times 10^{-6}$ . Three phenotypes are generated from different cohorts with different sample sizes 1000 , 500 , and 100 , respectively. The heritability for each phenotype is 0.01 . The overlapping individuals in all cohorts were 50 . Four simulated scenarios are created: in scenario **(A)**, the phenotype correlation between any pair of phenotypes is set at 0.1 ; in scenario **(B)**, it is raised to 0.2 ; Scenario **(C)** features a phenotype correlation of 0.5 ; and in scenario **(D)**, the phenotype correlation is set at 1 .

**FIGURE 2:**
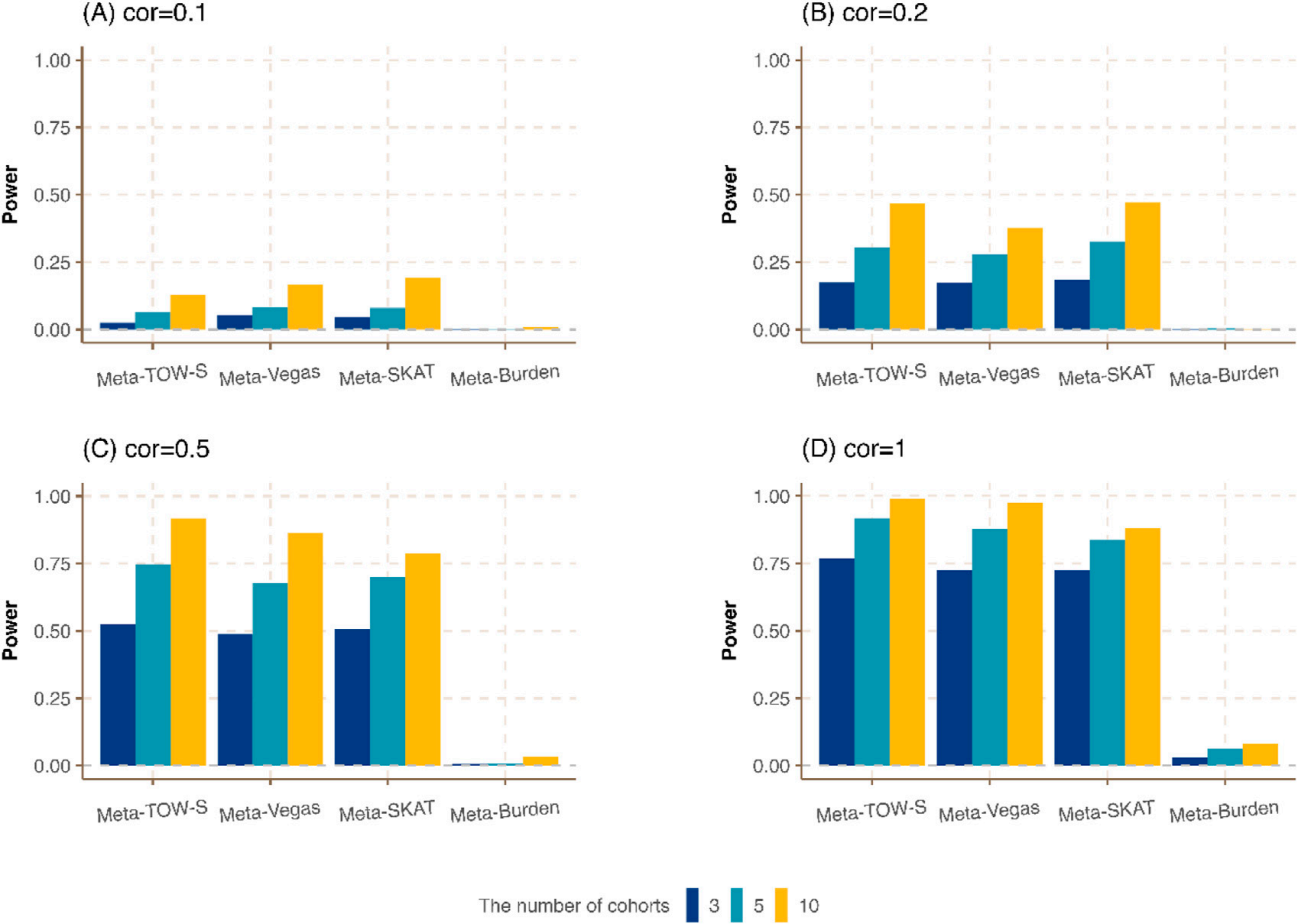
Power comparison of meta-analysis with different numbers of cohorts. 3 , 5 , and 10 phenotypes from different cohorts are generated, and the genetic heritability is fixed at 0.01 and is equally distributed among all causal variants in a region. All cohorts have the same sample size 200 . The overlapping sample size is 100 for all cohorts and the correlations between any pair of phenotypes in **(A–D)** are 0.1,0.2,0.5 , and 1 , respectively.

**FIGURE 3:**
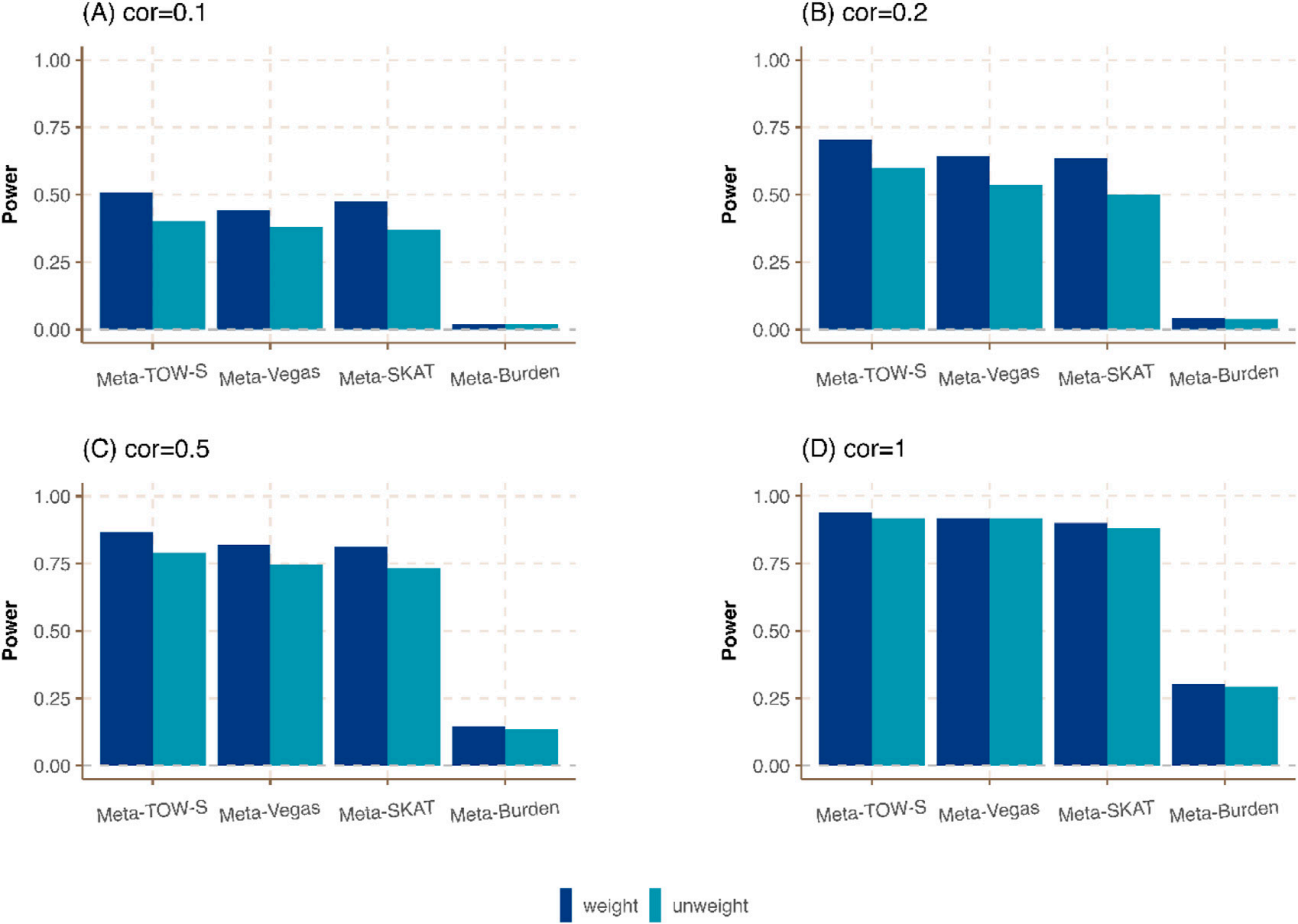
Power comparison of meta-analysis in the presence of weighting and unweighting scheme in Cauchy combination. Three phenotypes from three cohorts are generated. The sample sizes of these three cohorts are 2000,500 , and 100 , respectively. The heritability is fixed at 0.01 and is equally distributed among all causal variants in a region. The overlapping sample size is 10 among all cohorts and the correlations between any pair of phenotypes in **(A–D)** are 0.1,0.2,0.5, and 1 , respectively.

**FIGURE 4:**
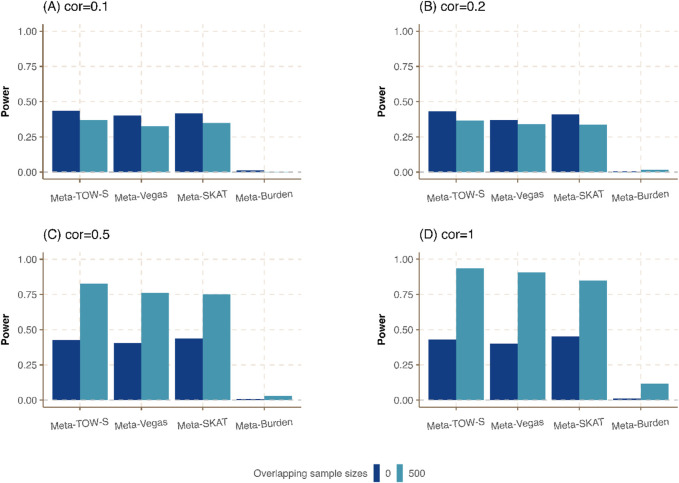
Power comparison of meta-analysis in the presence of the same sample size in each cohort but different overlapping individuals. The sample size of each cohort is 500 and the heritability for each phenotype is 0.01 . The overlapping sample sizes are 0 and 500 , and the correlations between any pair of phenotypes in **(A–D)** are 0.1,0.2,0.5, and 1 , respectively.

We compare the empirical power of Meta-TOW-S with Meta-Vegas, Meta-SKAT, and Meta-Burden. The power is defined as the proportion of test statistics with *p*-values less than the nominal significance level and we evaluate power at the nominal significance level $\alpha =2.5\times 10^{-6}$ after Bonferroni correction. For power comparisons, we generate phenotypes under the alternative hypothesis where the genetic effect is added correspondingly. Under each simulation setting, we generate 10,000 datasets to evaluate power at the nominal significance level $\alpha =2.5\times 10^{-6}$ . For the overlapping individuals, the phenotypic correlation between any pair of phenotypes across four scenarios: 0.1,0.2,0.5 , and 1 . A correlation of 1 indicates a simulation of mimicking one phenotype but from different cohorts. The primary simulations explore four distinct schemes. The first scheme compares the set-based association test for multiple phenotypes with that for a single phenotype. The second scheme evaluates the set-based association test for multiple phenotypes across varying numbers of traits, which are K=3,K=5 and K=10 . The third scheme tests the performance of a weighting scheme we designed within the Cauchy combination. The final scheme evaluates the set-based multiple phenotypes association test under different degrees of sample overlap. For simplicity, we set the heritability for each phenotype in the above scheme to be either $h_{k}^{2}=0.01$ or $h_{k}^{2}=0.3$ for k=1,…,K . The simulation results are depicted in Figures 1–4 for a heritability of 0.01 , while results for a heritability of 0.3 are presented in the Supplementary Material.

We first compare the meta-analysis with single phenotype-based set-level tests. It shows that the integration of different cohorts to detect the genetic variants in a region associated with at least one phenotype could boost power (Figure 1; Supplementary Figure S1). Our proposed Meta-TOW-S has slightly better performance compared with Meta-Vegas, and Meta-SKAT, and is better performed than Meta-Burden since the Burden-based method has poor performance if the effects of causal variants are in different directions. We also found the increase in correlation between pairs of phenotypes will boost power as well, indicating that the integration of phenotypes will benefit to detect more pleiotropic genes.

Next, we vary the number of cohorts where each cohort has the same sample size 200 and genetic heritability $h^{2}=0.01$ . Specifically, we create datasets with 3 , 5 , and 10 cohorts and assess the effect of incorporating multiple cohorts in the meta-analysis for the identification of pleiotropic genes. As illustrated in Figure 2; Supplementary Figure S2, Meta-TOW-S outperforms or performs equivalently well as the other three methods. It also shows that the increase in the number of cohorts boosts the power of all methods.

In Meta-TOW-S, Meta-Vegas, Meta-SKAT, and Meta-Burden, more weight is assigned to a large study, and a small weight is assigned to a small study in the Cauchy combination. We compare those four methods with the unweighting scheme where the weight is the same among all studies in the Cauchy combination. Figure 3; Supplementary Figure S3 shows that the weighting scheme-based meta-analysis has a higher power compared with an unweighting scheme. Specifically, when the correlation between any pair of phenotypes is low, the weighting scheme meta-analysis exhibits significantly enhanced statistical power. Conversely, when the correlation between any pair of phenotypes is high, there is only a slight improvement in power. It indicates that the weighting scheme has a slight improvement when the genetic effect contributes to all three studies equivalently if the phenotype correlation is 1 , which represents homogeneity across cohorts for the same phenotype.

Last, we consider the situation in which the sample sizes are the same in each cohort but different proportions of overlapping individuals are shared among all cohorts. As expected in Figure 4; Supplementary Figure S4, Meta-TOW-S outperforms the other three methods, and Meta-Vegas and Meta-SKAT have comparable performance. It shows that the power is further improved when there are larger overlapping samples between studies if phenotypes are highly correlated, which could be attributed to reduced heterogeneity across cohorts.

### 4.3 Real data analysis

**FIGURE 5:**
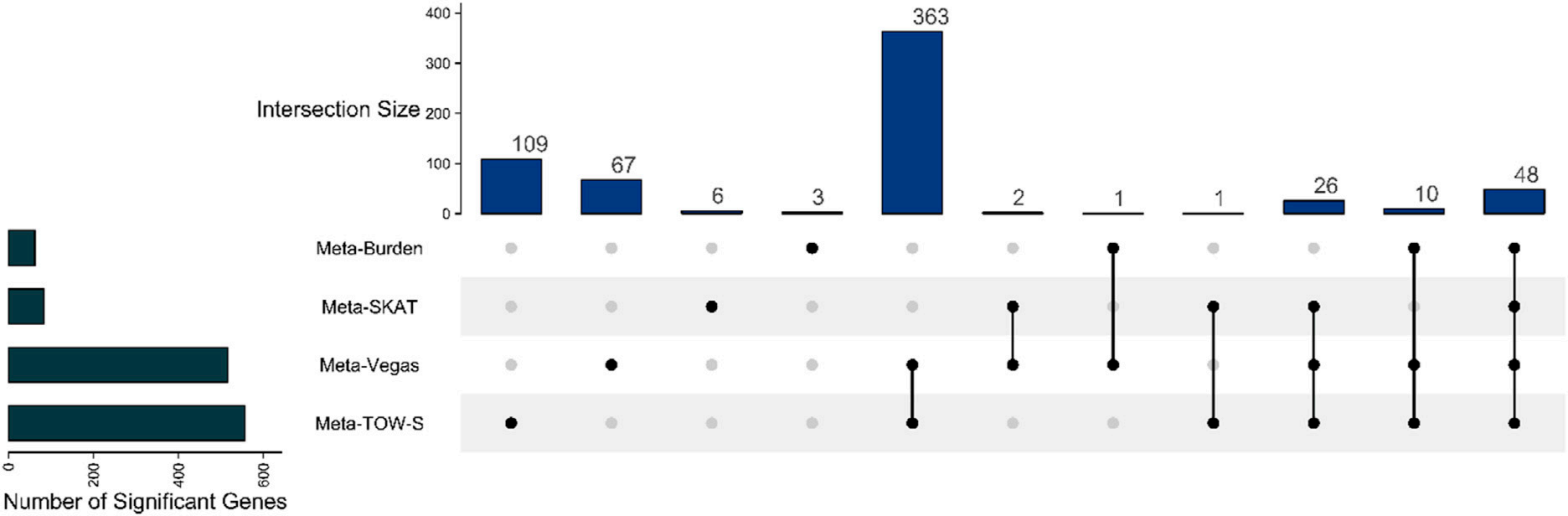
Upset plot showing the number of overlapping detected genes between Meta-TOW-S, Meta-Vegas, Meta-SKAT, and Meta-Burden.

We apply these four methods to GWAS summary statistics for four psychiatric disorders available from the Psychiatric Genomics Consortium (PGC) (Sullivan et al., 2018). These phenotypes are attention-deficit/hyperactivity disorder (ADHD), autism spectrum disorder (ASD), bipolar disorder (BD), and schizophrenia (SCZ) (Zhang et al., 2021). The sample sizes for these four traits range from 46,351 to 105,318 , with all individuals of European ancestry. We utilize the LD structure data from the 1,000 Genome Project Phase III European population as the reference in set-based multiple phenotype association test. The details of these four GWAS summary statistics are summarized in Supplementary Table S1. The analysis of these four methods for the four psychiatric disorders is summarized using the UpSet plot shown in Figure 5. We use the gene-based GWAS significance level $\alpha =2.5\times 10^{-6}$ in the analysis. For Meta-Burden and Meta-SKAT, the weight in the gene-based tests are defined in relation to the minor allele frequency (MAF) of genetic varaints. Specifically, we use the default weight $w_{j}=1/\sqrt{MAF_{j}\left(1-MAF_{j}\right)}$ for Meta-Burden and $w_{j}=Beta\left(MAF_{j};a_{1},a_{2}\right)$ for Meta-SKAT, where the beta distribution density function has pre-specified parameters $a_{1}$ , $a_{2}$ , and $MAF_{j}$ of the $j^{th}$ variant. As a result, there are 557 genes detected by Meta-TOW-S, 517 genes detected by Meta-Vegas, 83 genes detected by Meta-SKAT, and 62 genes detected by Meta-Burden. These results indicate that Meta-TOW-S identifies more significant genes compared to Meta-Vegas, Meta-SKAT, and Meta-Burden.

### 4.4 Enrichment analysis

We implement the Gene Set Enrichment Analysis (GSEA) to analyze the significant genes identified by Meta-TOW-S that are enriched toward the top list of genes that are associated with specific biological pathways, processes, functions, or diseases (Subramanian et al., 2005). Gene Ontology (GO) is a community-based bioinformatics resource that employs ontologies to represent biological knowledge and describes information about gene and gene product information (Peng et al., 2017). Go is widely used to infer functional information for gene products, such as gene function enrichment, protein function prediction, and disease association analysis. And Go contains three categories: cellular component (CC), molecular function (MF: the biological function of the gene), and biological process (BP: pathways or larger processes that multiple gene products are involved in). KEGG (Kyoto Encyclopedia of Genes and Genomes) is a database resource for understanding high-level functions and utilities of the biological system. KEGG is used to search for the pathways associated with the identified genes. Detection of KEGG pathway database over-representation against a universal Homo Sapien background is assessed by hypergeometric tests (Solomon et al., 2022; Kanehisa et al., 2016). DisGeNET is an integrative and comprehensive resource of gene-disease associations from several public data sources and the literature (Piñero et al., 2015; Yu et al., 2014). It contains gene-disease associations and variant-gene-disease associations. The disease enrichment analysis is used to assess whether the genes associated with multiple phenotypes in meta-analysis are overrepresented in specific gene sets. A Bonferroni corrected cutoff of 0.05 was used for the significance of the pathway and disease. For genes annotation, we employed the org.Hs.e.g.,.db package in R. This package offers an extensive set of annotations for the human genome, including mappings between different gene identifiers and detailed genomic features.

**FIGURE 6:**
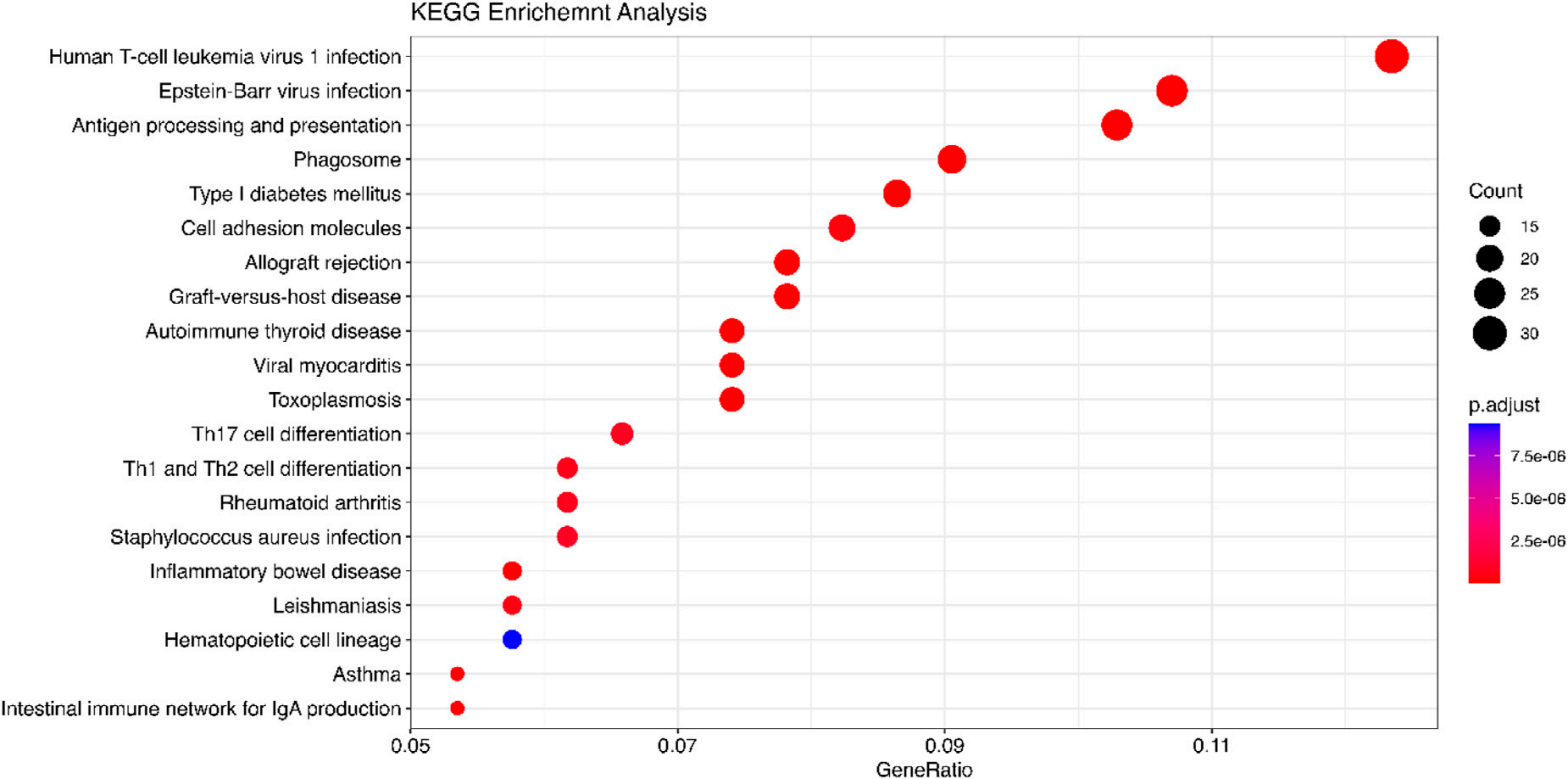
KEGG analysis is performed using the DOSE package. The significant genes are detected by Meta-TOW-S. The size of the circles represents the number of differential genes in a pathway. GeneRatio is the ratio of the number of differentially expressed genes annotated in a pathway to the number of all genes annotated in these pathways.

**FIGURE 7:**
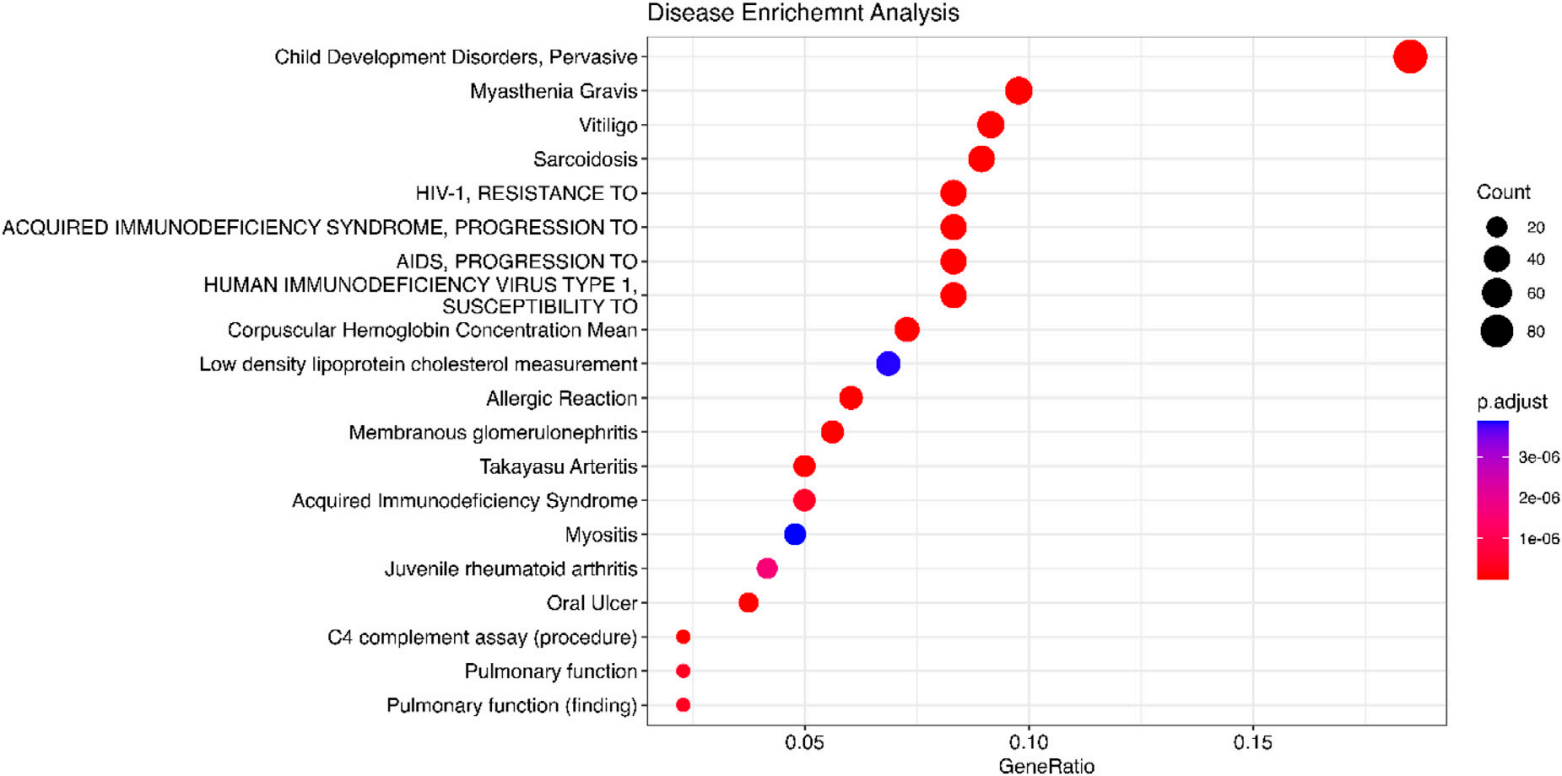
Disease enrichment analysis is performed using DisGeNET. The significant genes are detected by Meta-TOW-S.

We use the 557 significant genes identified by Meta-TOW-S, which are associated with four psychiatric disorders, for the enrichment analysis. We perform enrichment analyses at two different levels: pathways and diseases (Figures 6 and 7; Supplementary Figure S5). The top 20 enriched KEGG pathways are summarised in Figure 6 with results sorted from lowest to highest *p*-values. Of the 280 KEGG pathways tested, 37 were statistically significant after adjusting for multiple testing. The top KEGG pathways predominantly belong to groups associated with Human T-cell Leukemia virus 1 infection, Epstein-Barr virus infection, Antigen processing and presentation, etc. In the disease enrichment analysis, of the 5,496 disease tests, 455 had Bonferroni-corrected enrichment *p*-values lower than 0.05. The implicated genes are involved in Child Development Disorders Pervasive, Myasthenia Gravis, Vitiligo, Sarcoidosis, etc. (Figure 7).

## 5 Discussion

Much research suggests that many genes are associated with multiple correlated or even distinct phenotypes, and such associations have been termed cross-phenotype associations, which is relevant to pleiotropy in complex phenotypes (Li and Zhu, 2017). We propose a new method, Meta-TOW-S, which integrates association evidence of multiple phenotypes from study-specific GWAS summary statistics and thus detects the significant pleiotropic genes. This method Meta-TOW-S is based on the set-based test which uses the weights that maximize the score test statistic to increase power. To combine the test statistics from multiple GWAS cohorts, Meta-TOW-S uses the Cauchy combination by assigning more weight to a large GWAS to account for more biological information. In addition, Meta-TOW-S enables analysis across multiple phenotypes by accommodating overlapping samples from different cohorts.

To mimic real multiple comprehensive study-specific phenotypes, we develop a phenotype simulator that encompasses a simulation scheme capable of modeling multiple phenotypes with multiple underlying genetic loci, intricating noise structures, and different correlation patterns among the phenotypes with overlapped samples across different cohort studies. Our simulations show that the Type I error rates of Meta-TOW-S are well maintained under different conditions of phenotype correlation structures and overlapping samples and are more powerful than the other three comparison methods under most scenarios. We also find that the power of meta-analysis is significantly increased compared to the single phenotype set-based tests. A higher phenotype correlation, larger overlapping samples across multiple cohort studies, and more cohorts can increase power as well. We apply Meta-TOW-S to the summary statistics of four psychiatric disorders provided by the Psychiatric Genomics Consortium (PGC): attention-deficit/hyperactivity disorder (ADHD), autism spectrum disorder (ASD), bipolar disorder (BD), and schizophrenia (SCZ). As a result, 557 significant cross-phenotype associations are identified by Meta-TOW-S which is more than the number of genes identified by the other three methods. In the enrichment analysis, the gene sets of Child Development Disorders Pervasive are more enriched for genes associated with these four psychiatric disorders. In the KEGG pathway analysis, the significant genes identified by Meta-TOW-S showed notable enrichment in immune-related pathways rather than neurological processes. However, it is important to recognize that there is substantial evidence linking immune system involvement to neurological disorders. For instance, research has indicated that developmental disorders, such as Autism Spectrum Disorders (ASDs), can involve significant immune activity, including neuroinflammation, which plays a crucial role in the pathophysiology of these conditions (Vargas et al., 2005; Ashwood and Van de Water, 2004). Additionally, studies have demonstrated that elevated levels of regulatory T cells are associated with an increased risk of Attention-Deficit/Hyperactivity Disorder (ADHD) (Çetin et al., 2022), suggesting that immune dysregulation may contribute to the manifestation of neurological symptoms in certain contexts.

Meta-TOW-S has multiple advantages for identifying cross-phenotype associations. First of all, Meta-TOW-S can integrate information from multiple cohort studies to increase power and has the potential to detect more pleiotropic genes. Secondly, the test statistic has a standard Cauchy distribution under the null hypothesis and greatly reduces the computing time. Thirdly, this method is based on GWAS summary statistics from different cohort studies and GWAS summary statistics are more accessible than individual-level phenotype and genotype data The last point is that Meta-TOW-S gives more weight to the study with a larger sample size. Meanwhile, the developed phenotypes simulator can mimic complex structures in a meta-analysis which can be applied in other cross-phenotype analyses. However, Meta-TOW-S leverages information from correlated phenotypes to enhance its statistical power. Simulation results demonstrate that Meta-TOW-S surpasses other methods in power performance when phenotypic correlations are strong. However, when the correlation between phenotypes is weak, Meta-TOW-S may not outperform other methods, as it does not rely heavily on borrowing information from other phenotypes in such scenarios.

Currently, the framework of Meta-TOW-S needs to estimate the correlation matrix from a reference panel due to the unavailability of individual genotype data. However, the choice of the reference panel may influence the performance of Meta-TOW-S. The second challenge is that we use a correlation pruning procedure to ensure that the correlation matrix is fully ranked, which may drop some correlated variants in a gene.

In summary, the Meta-TOW-S method is a very useful method for detecting gene associations of multiple phenotypes from different GWAS cohorts. Meta-TOW-S has robust power and can handle different scenarios such as diverse phenotype correlation, and intricating cohort studies. The computational efficiency of Meta-TOW-S can also improve genetic discovery for hundreds of phenotypes across multiple GWAS cohorts in compliance with data privacy.

## Data Availability

The original contributions presented in the study are included in the article/Supplementary Material, further inquiries can be directed to the corresponding author.
