# Supplementary material for: Meta-analysis of set-based multiple phenotype association test based on GWAS summary statistics from different cohorts
Source: Front Genet. 2024 Sep 5;15:1359591. doi: 10.3389/fgene.2024.1359591 (PMC11410627; doi:10.3389/fgene.2024.1359591)
Supplement: Supplementary file 1 [file DataSheet2.PDF]

Supplementary Materials

Meta-analysis of set-based multiple phenotype association test based on GWAS summary statistics from different cohorts

Lirong Zhu[1], Shuanglin Zhang[1], Qiuying Sha[1,*]

[1]Department of Mathematical Sciences, Michigan Technological University, Houghton, Michigan, USA

[*]Corresponding author: Qiuying Sha, Department of Mathematical Sciences, Michigan Technological University, Houghton, Michigan 49931, USA. E-mail: qsha@mtu.edu

Supplemental text

Lemma: Under this model, the phenotypic correlation for cohort i and cohort j among the N_s overlapping samples is $\rho_{ij} = \rho_{g_{ij}} + \rho_{e_{ij}}$.

Proof: Let G denote an $1 \times M$ vector of standardized genotypes for an arbitrary individual. Thus, the phenotype covariance between phenotype 1 and phenotype 2 by the law of total covariance is

$$\begin{aligned} Cov(y_1, y_2) &= E\left[Cov(y_1, y_2 \mid G)\right] + Cov\left[E(y_1 \mid G), E(y_2 \mid G)\right] \\ &= E\left[Cov\left(\sum_j G_j \beta_{1j} + \varepsilon_1,\ \sum_j G_j \beta_{2j} + \varepsilon_2 \mid G\right)\right] \end{aligned}$$

where the second line follows from the fact that $E(y_1 \mid G) = 0, E(y_2 \mid G) = 0$, irrespective of G. First,

$$\begin{aligned} &Cov\left(\sum_j G_j \beta_{1j} + \varepsilon_1,\ \sum_j G_j \beta_{2j} + \varepsilon_2 \mid G\right) \\ &= E\left[\left(\sum_j G_j \beta_{1j} + \varepsilon_1,\right)\left(\sum_j G_j \beta_{2j} + \varepsilon_2\right)\right] \\ &= E\left[\sum_j G_j^2 \beta_{1j} \beta_{2j}\right] + E\left[\varepsilon_1 \varepsilon_2\right] \\ &= \sum_j G_j^2 \frac{\rho_{g_{12}}}{M} + \rho_{e_{12}} \end{aligned}$$

Thus,

$$\begin{aligned} Cov(y_1, y_2) &= E\left[Cov\left(\sum_j G_j \beta_{1j} + \varepsilon_1,\ \sum_j G_j \beta_{2j} + \varepsilon_2 \mid G\right)\right] \\ &= E\left[\sum_j G_j^2 \frac{\rho_{g_{12}}}{M} + \rho_{e_{12}}\right] \\ &= \rho_{g_{12}} + \rho_{e_{12}} = \rho_{12} \end{aligned}$$

The correlation between two phenotypes is ρ_{12} since the variance of phenotype is 1.

Supplemental tables:

Table S1. GWAS summary statistics of the four psychiatric disorders.

Trait Name	Abbreviation	N	M	Ref
Attention-Deficit/Hyperactivity Disorder	ADHD	53293	30478444	(Demontis et al., 2019)
Autism Spectrum Disorder	ASD	46351	30804558	(Grove et al. 2019)
Bipolar Disorder	BD	51710	31043756	(Stahl et al. 2019)
Schizophrenia	SCZ	105318	29483656	(Pardiñas et al., 2018)

Note: N is the sample size of original GWASs; M is the total number of variants across the whole genome;

Supplemental figures:

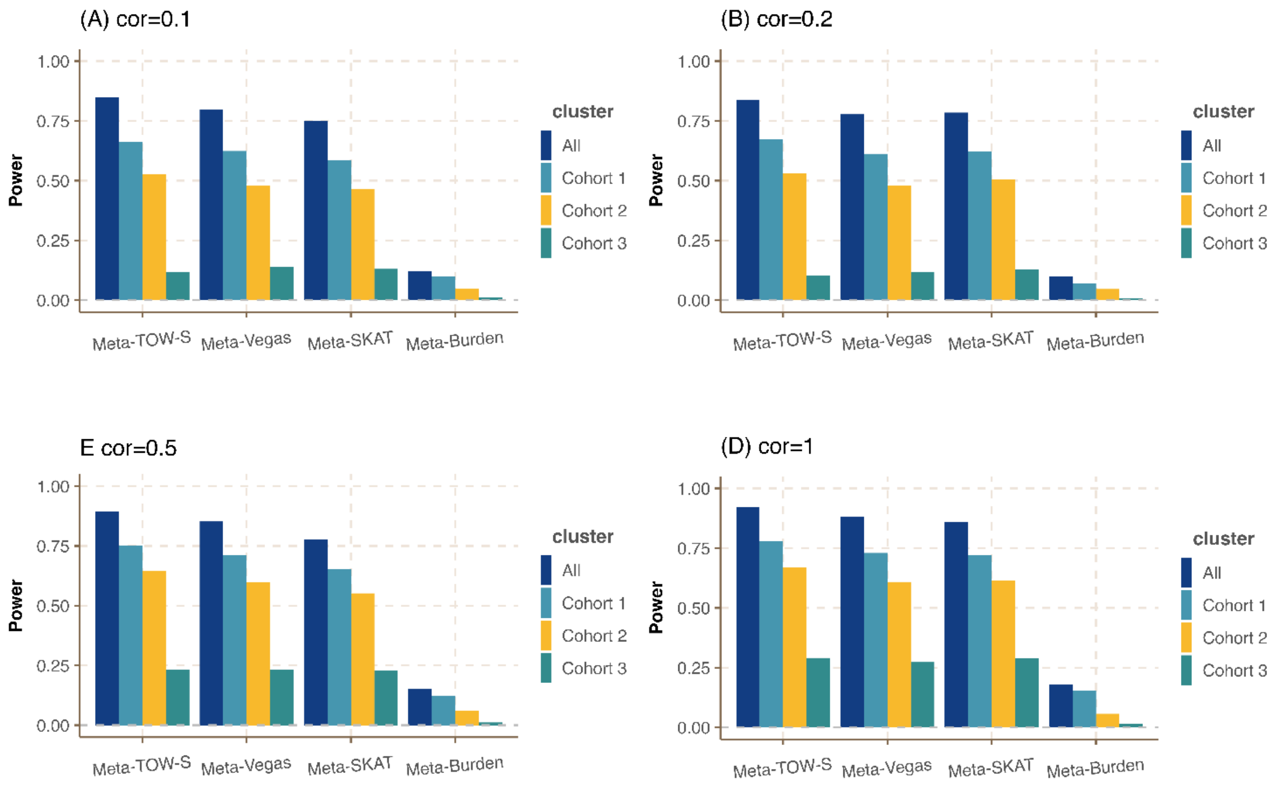

Figure S1: Power comparison of Meta-TOW-S, Meta-Vegas, Meta-SKAT, and Meta-Burden with single phenotype-based set-level tests at the significance level $\alpha = 2.5 \times 10^{-6}$. Three phenotypes are generated from different cohorts with different sample sizes 1000, 500, and 100, respectively. The heritability for each phenotype is 0.3. The overlapping individuals in all cohorts were 50. Four simulated scenarios are created: in scenario (A), the phenotype correlation between any pair of phenotypes is set at 0.1; in scenario (B), it is raised to 0.2; Scenario (C) features a phenotype correlation of 0.5; and in scenario (D), the phenotype correlation is set at 1.

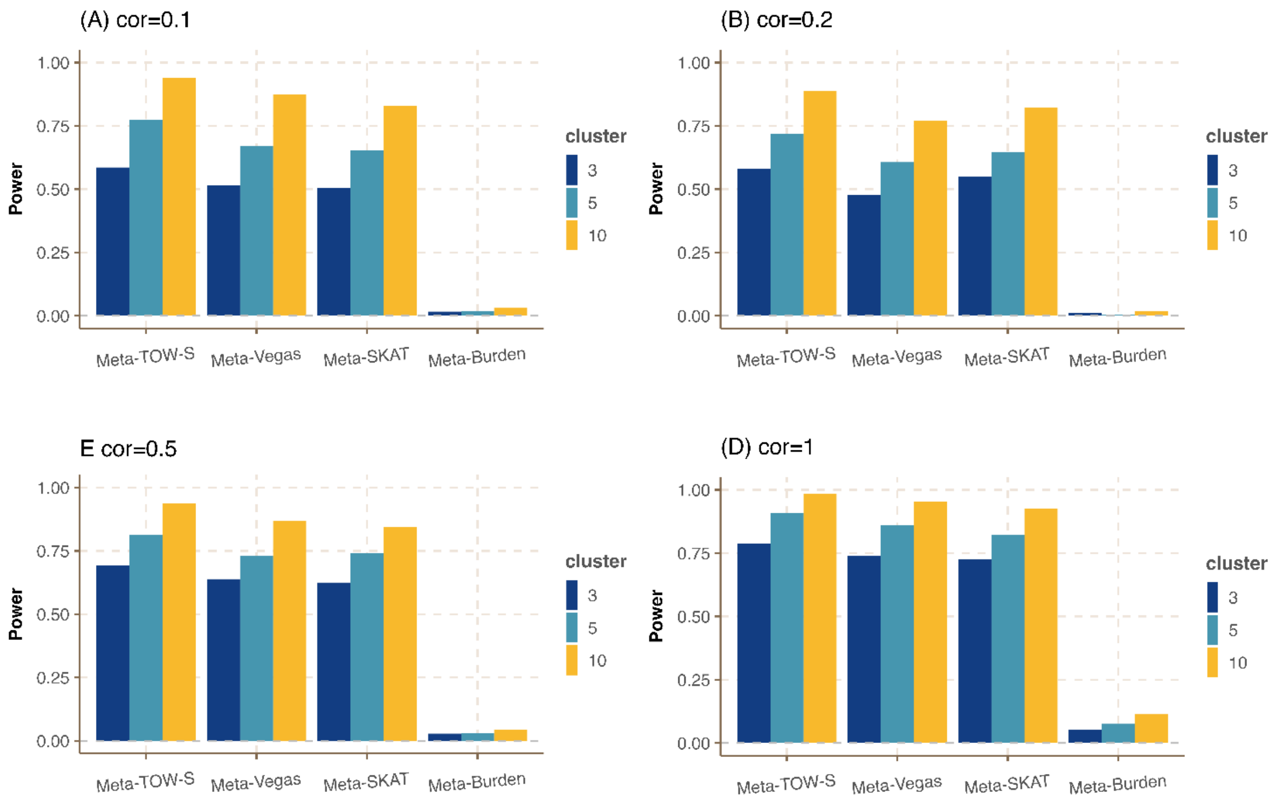

Figure S2: Power comparison of meta-analysis with different numbers of cohorts. 3, 5, and 10 phenotypes from different cohorts are generated, and the genetic heritability is fixed at 0.3 and is equally distributed among all variants in a region. All cohorts have the same sample size 200. The overlapping sample size is 100 for all cohorts and the correlations between any pair of phenotypes in (A) - (D) are $0.1, 0.2, 0.5$, and 1, respectively.

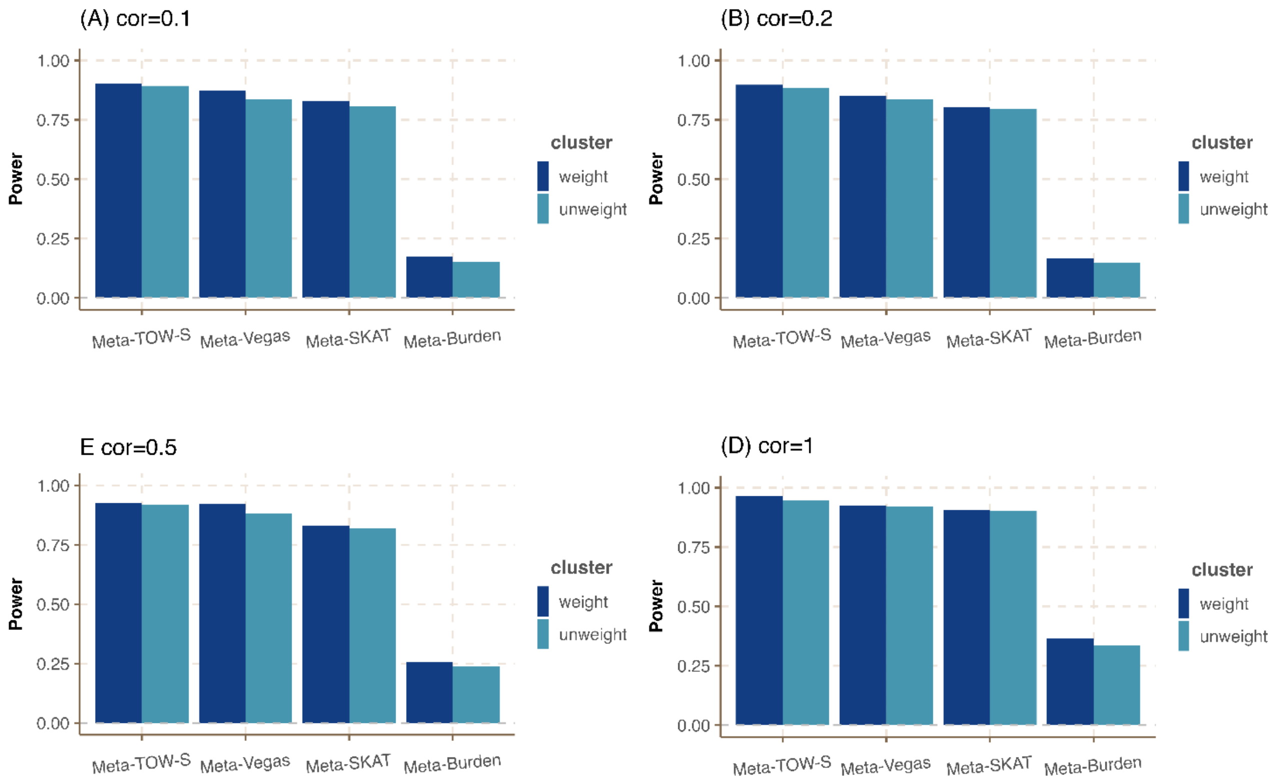

Figure S3: Power comparison of meta-analysis in the presence of weighting and unweighting scheme in Cauchy combination. Three phenotypes from three cohorts are generated. The sample sizes of these three cohorts are $2000, 500$, and 100, respectively. The heritability is fixed at 0.3 and is equally distributed among all causal variants in a region. The overlapping sample size is 10 among all cohorts and the correlations between any pair of phenotypes in (A) - (D) are $0.1, 0.2, 0.5$, and 1, respectively.

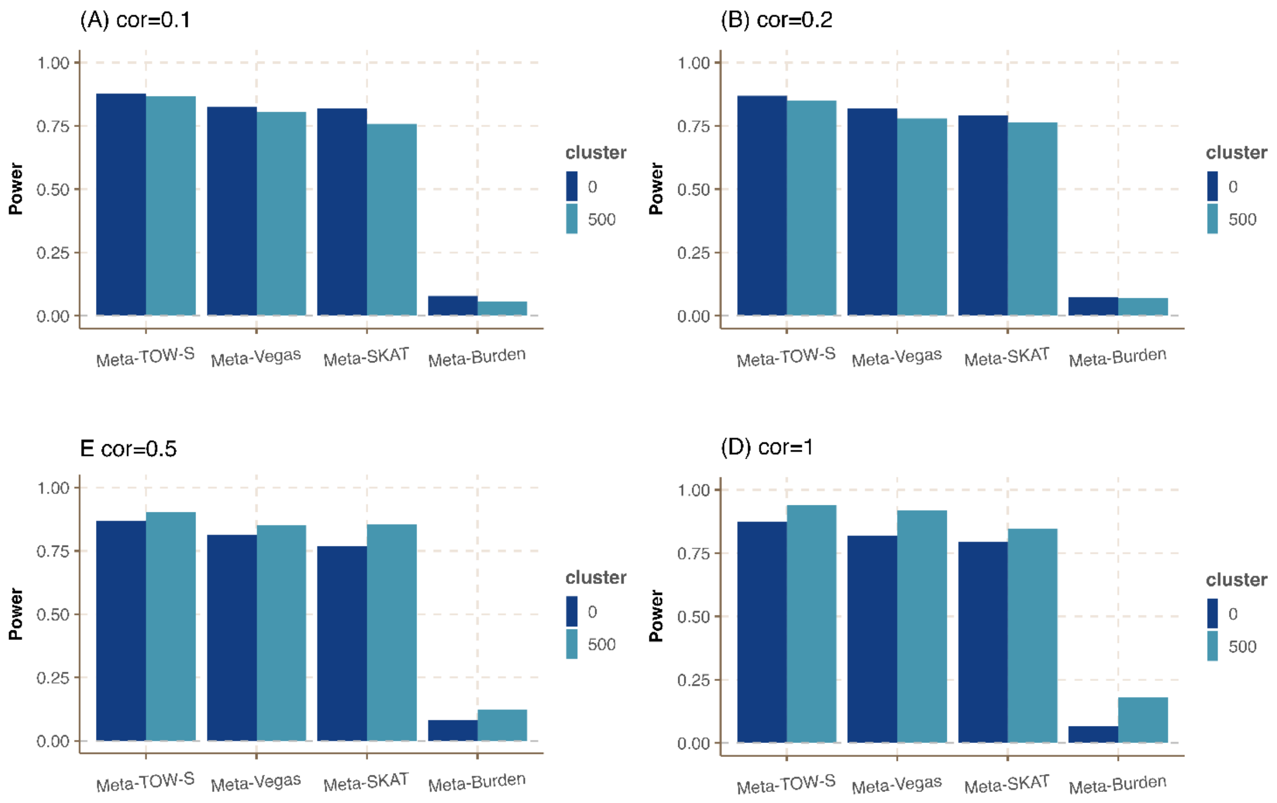

Figure S4: Power comparison of meta-analysis in the presence of the same sample size in each cohort but different overlapping individuals. The sample size of each cohort is 500 and the heritability for each phenotype is 0.3. The overlapping sample sizes are 0 and 500, and the correlations between any pair of phenotypes in (A) - (D) are $0.1, 0.2, 0.5,$ and 1, respectively.

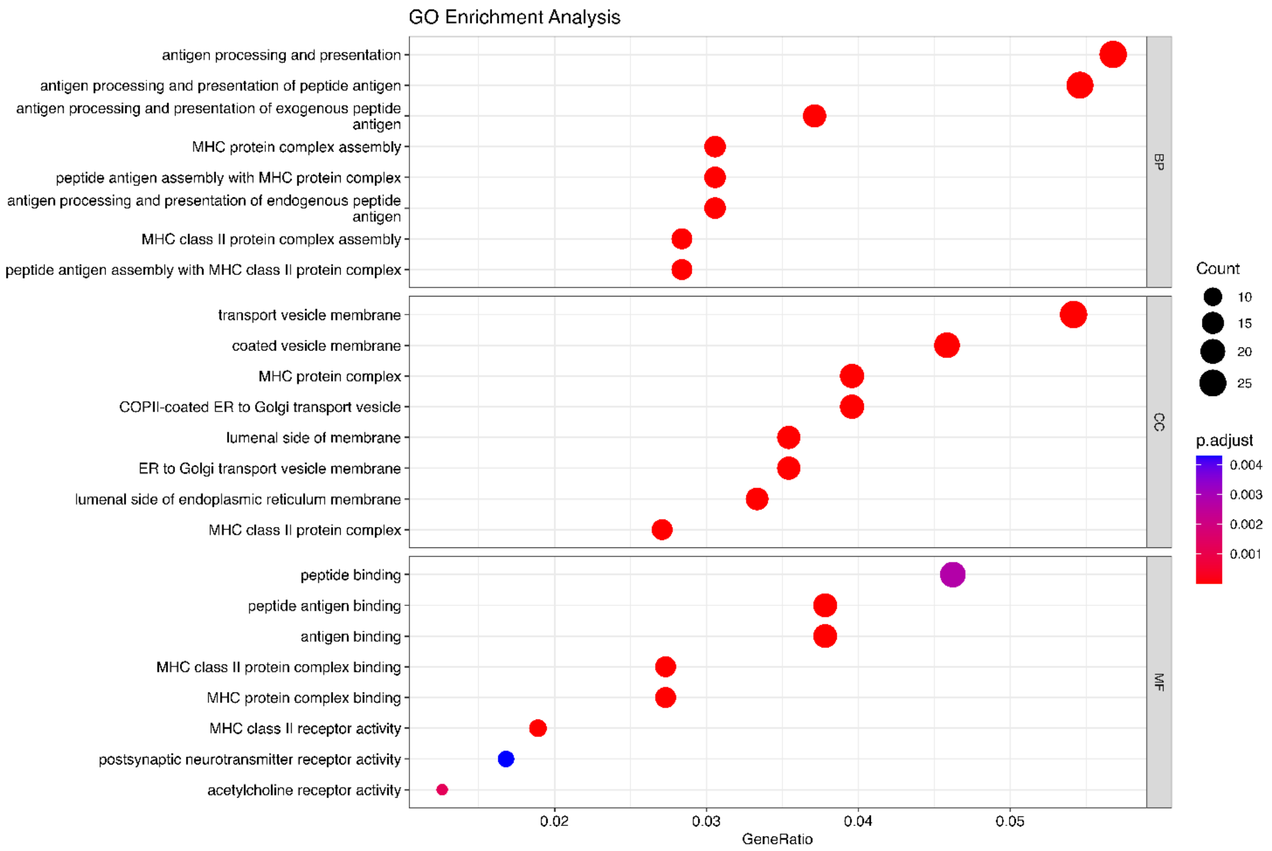

Figure S5: GO analysis using DOSE package for the identification of the enrichment functions of three independent categories of genes: biological process (BP), cellular component (CC), and molecular function (MF).
